# Distribution of indole-3-acetic acid in *Petunia hybrida* shoot tip cuttings and relationship between auxin transport, carbohydrate metabolism and adventitious root formation

**DOI:** 10.1007/s00425-013-1907-z

**Published:** 2013-06-14

**Authors:** Amir H. Ahkami, Michael Melzer, Mohammad R. Ghaffari, Stephan Pollmann, Majid Ghorbani Javid, Fahimeh Shahinnia, Mohammad R. Hajirezaei, Uwe Druege

**Affiliations:** 1 Institute of Biological Chemistry (IBC), Washington State University, Pullman, WA 99164-6340 USA; 2 Leibniz Institute of Plant Genetics and Crop Plant Research (IPK), Corrensstr. 3, Gatersleben, 06466 Seeland, Germany; 3 Parque Científico y Tecnológico de la U.P.M, Centro de Biotecnología y Genómica de Plantas U.P.M.-I.N.I.A, Campus de Montegancedo, Pozuelo de Alarcón, 28223 Madrid, Spain; 4 Department of Agronomy and Plant Breeding Sciences, College of Abureihan, University of Tehran, Tehran, Iran; 5 Australian Centre for Plant Functional Genomics, University of Adelaide, Waite Campus, Hartley Grove Urrbrae, Adelaide, 5064 Australia; 6 Leibniz Institute of Vegetable and Ornamental Crops Großbeeren/Erfurt e.V. (IGZ), Kuehnhaeuser Str. 101, 99090 Erfurt, Germany

**Keywords:** Polar auxin transport (PAT), IAA, *GH3*, Sink establishment, Petunia, Root development

## Abstract

To determine the contribution of polar auxin transport (PAT) to auxin accumulation and to adventitious root (AR) formation in the stem base of *Petunia* *hybrida* shoot tip cuttings, the level of indole-3-acetic acid (IAA) was monitored in non-treated cuttings and cuttings treated with the auxin transport blocker naphthylphthalamic acid (NPA) and was complemented with precise anatomical studies. The temporal course of carbohydrates, amino acids and activities of controlling enzymes was also investigated. Analysis of initial spatial IAA distribution in the cuttings revealed that approximately 40 and 10 % of the total IAA pool was present in the leaves and the stem base as rooting zone, respectively. A negative correlation existed between leaf size and IAA concentration. After excision of cuttings, IAA showed an early increase in the stem base with two peaks at 2 and 24 h post excision and, thereafter, a decline to low levels. This was mirrored by the expression pattern of the auxin-responsive *GH3* gene. NPA treatment completely suppressed the 24-h peak of IAA and severely inhibited root formation. It also reduced activities of cell wall and vacuolar invertases in the early phase of AR formation and inhibited the rise of activities of glucose-6-phosphate dehydrogenase and phosphofructokinase during later stages. We propose a model in which spontaneous AR formation in *Petunia* cuttings is dependent on PAT and on the resulting 24-h peak of IAA in the rooting zone, where it induces early cellular events and also stimulates sink establishment. Subsequent root development stimulates glycolysis and the pentose phosphate pathway.

**Electronic supplementary material:**

The online version of this article (doi:10.1007/s00425-013-1907-z) contains supplementary material, which is available to authorized users.

## Introduction

When a cutting is removed from a plant under appropriate conditions, it may produce a new root system and finally an entire individual with a balanced root-to-shoot ratio (Lovell and White 1987). These events involve various anatomical, physiological and molecular changes associated with wound responses in addition to those involved in root formation itself. Adventitious rooting can be considered as an interesting process of post-embryonic organogenesis as it describes the development of new root tissues in locations other than the primary root system (Blakesley et al. 1991). In addition, adventitious root (AR) formation in leafy stem cuttings is a crucial physiological process for the propagation of many ornamental plant species. Despite intensive control of environmental factors in the modern propagation industry, high economic losses still occur as a result of insufficient rooting, which is in direct contrast with the increasing demand for fast and synchronous rooting to meet horticultural standards (Druege 2009).

The formation of ARs is a complex process. It involves successive developmental phases requiring different hormonal signals and other endogenous factors, in which auxin plays a pivotal role (Kevers et al. 1997; De Klerk et al. 1999), and is affected by environmental factors, such as wounding or light (Sorin et al. 2005). Auxins, of which indole-3-acetic acid (IAA) constitutes the most important endogenous physiologically active fraction (Kerr and Bennett 2007), have been shown to be effective inducers of AR initiation (Ludwig-Müller 2009). Since their discovery, they have been frequently used in horticultural practice to stimulate rooting and particularly in plant species that show only weak AR formation without external stimuli (Hartmann et al. 2011).

Considering auxin homeostasis in the rooting zone, it has been concluded from observations on different plant species that high concentrations of free auxin are needed during the induction phase of adventitious rooting, whereas during later stages, high auxin levels obviously have an inhibitory action on differentiation and outgrowth of root primordia (De Klerk et al. 1999). One way to reduce active auxin is degradation via oxidative decarboxylation, which is probably associated with one of the important functions of higher peroxidase activity repeatedly observed after the induction phase (Kevers et al. 1997; Tonon et al. 2001). Another way to reduce the level of active auxin is by conjugation to sugars and amino acids (Woodward and Bartel 2005), which may be indicated by an increased levels of IAA conjugates such as indole-3-acetylaspartic acid (IAAasp) during later stages of AR formation (Nordström and Eliasson 1991; Garcia Gomez et al. 1994). In this respect, auxin-inducible *GH3* (Gretchen Hagen 3) genes can play an important role in the control of free auxin levels because specific *GH3*s can catalyse conjugation of amino acids to IAA (Staswick et al. 2005; Wang et al. 2007). Because of the presence of auxin-responsive elements in the *GH3* s’ promoter region, the expression of *GH3* genes can be used to monitor auxin activity (Hagen et al. 1991; Wang et al. 2007).

Spontaneous AR formation, which does not rely on auxin application, is observed in leafy stem cuttings of many plant species in response to excision from the donor plant. Basipetal auxin transport is assumed to contribute to this phenomenon (Blakesley 1994; De Klerk et al. 1999). This conception is mainly based on the following observations. Firstly, monitoring of endogenous auxin, particularly of IAA, revealed a transient increase in the rooting zone (Blakesley et al. 1991; Blažková et al. 1997; Tonon et al. 2001). Secondly, labelled auxin applied to the apex of cuttings was transported to the stem base (Baadsmand and Andersen 1984; Guerrero et al. 1999). Finally, removal of potential source organs of auxin or application of blockers of polar auxin transport (PAT), such as naphthylphthalamic acid (NPA) or triiodobenzoic acid (TIBA), decreased AR formation (Liu and Reid 1992; Garrido et al. 2002). For example, decapitation and treatment of pea stem cuttings with NPA led to the reduction in IAA levels in cutting bases during the first days after excision, which was associated with lower numbers and shorter lengths of ARs (Nordström and Eliasson 1991; Koukourikou-Petridou and Bangerth 1997). Similarly, application of TIBA to avocado cuttings inhibited the differentiation of root primordia and reduced the percentage of rooted cuttings, while the IAA level in the basal stem was only slightly reduced (Garcia Gomez et al. 1994). These studies did not demonstrate a significant increase of IAA in the stem base of non-treated control cuttings, although they did produce a high number of roots. Because a transient increase in the level of IAAasp was detected in the basal part of untreated cuttings, the authors speculated that the initial IAA level could be sufficient to induce ARs or that a steady but non-detected release of IAA from IAAasp possibly contributed to AR formation (Nordström and Eliasson 1991; Garcia Gomez et al. 1994). However, Blakesley et al. (1991) detected a sharp peak of IAA in hypocotyls of *Phaseolus aureus* already within the first 10-h post excision. Thus, the first samplings of pea and avocado at 24 h and 3 days post excision, respectively (Nordström and Eliasson 1991; Koukourikou-Petridou and Bangerth 1997; Garcia Gomez et al. 1994), may have missed the transient IAA peak. Overall, there are only a few studies that combine modifications of auxin transport with early and frequent analysis of the auxin level in the rooting zone and with precise anatomical investigation.

The role of auxin transport and accumulation in the rooting zone is particularly unclear in relation to the response of carbohydrate metabolism, frequently observed during AR formation in cuttings (Ahkami et al. 2009; Druege 2009). Interrelationships between auxin and carbohydrate metabolism during adventitious rooting have been investigated by the application of auxins such as α-naphthalene acetic acid and indole-3-butyric acid and monitoring of carbohydrate levels, carbon translocation and activities of some enzymes in the rooting zone. It has been found that auxin application stimulated mobilization of carbohydrates in the upper shoot, increased the translocation of assimilates and increased sugar availability at the site of root primordia development (Altman and Wareing 1975; Haissig 1986; Husen and Pal 2007; Agulló-Antón et al. 2011). Haissig (1974) observed a stimulation of activity of glycerin-aldehyde-3-phosphate dehydrogenase together with enhanced root primordium initiation in the rooting zone of bean hypocotyl cuttings after IAA treatment and suggested that carbohydrate utilization is also subject to auxin. Considering the response of carbohydrate and protein levels in the rooting zone of *Tectonia grandis* cuttings, Husen and Pal (2007) proposed that auxin contributes to the release of energy and mobilization of proteins, which are necessary for cell division and differentiation. Furthermore, there is increasing evidence that auxin homeostasis and auxin response of root development can be modulated by sugar signalling (Mishra et al. 2009). However, the complex interactions between auxin, overall primary metabolism and cell division during AR formation are far from being elucidated.

*Petunia hybrida* is an ornamental plant of high economic importance in global horticulture. Over the past two decades, the genus *Petunia* has served as an excellent model system for uncovering the molecular, biochemical and physiological bases of several plant processes (Underwood et al. 2009). Recently, we established *P. hybrida* as a model species to study molecular and physiological bottle-necks in adventitious rooting of leafy stem cuttings. Focusing on the response of primary metabolism in the rooting zone in relation to anatomical stages, we defined three metabolic phases of AR formation: (1) the sink establishment phase, characterized by apoplastic unloading of sucrose as reflected by induced expression and high activity of cell wall invertase, (2) the recovery phase, characterized by replenishment of resources and (3) the maintenance phase, in which a steady state is maintained via symplastic unloading of sucrose (Ahkami et al. 2009). Ahkami et al. (2009) also observed a fast and transient increase in jasmonic acid in the rooting zone and jasmonates has been shown to stimulate auxin biosynthesis, modulate PAT and interact with auxin signalling (Sun et al. 2009; Hoffmann et al. 2011). However, the role of auxin in AR formation of *P. hybrida* cuttings is still poorly understood. This particularly applies to the contribution of auxin transport and to the interrelationship between auxin and the metabolic response.

The objectives of the present study were as follows: (1) to determine the initial spatial distribution of IAA as an important physiologically active auxin fraction in *P. hybrida* cuttings, (2) to elucidate the relationship between auxin transport, temporal distribution of auxin in the rooting zone and spontaneous development of ARs in *P.* *hybrida* in response to excision from donor plants and (3) to investigate the possible impact of auxin transport on the primary metabolic responses during AR formation. The spatial and temporal distribution of IAA was analysed in the leaves and stem base of cuttings in non-treated and NPA-treated cuttings using gas chromatography–tandem mass spectroscopy (GC–MS/MS). These experiments were complemented with time course analysis of *GH3* expression, of metabolites, of activities of key enzymes and of anatomical changes during AR formation stages.

## Materials and methods

### Plant material, growth conditions, NPA treatment and sampling

**Fig. 1:**
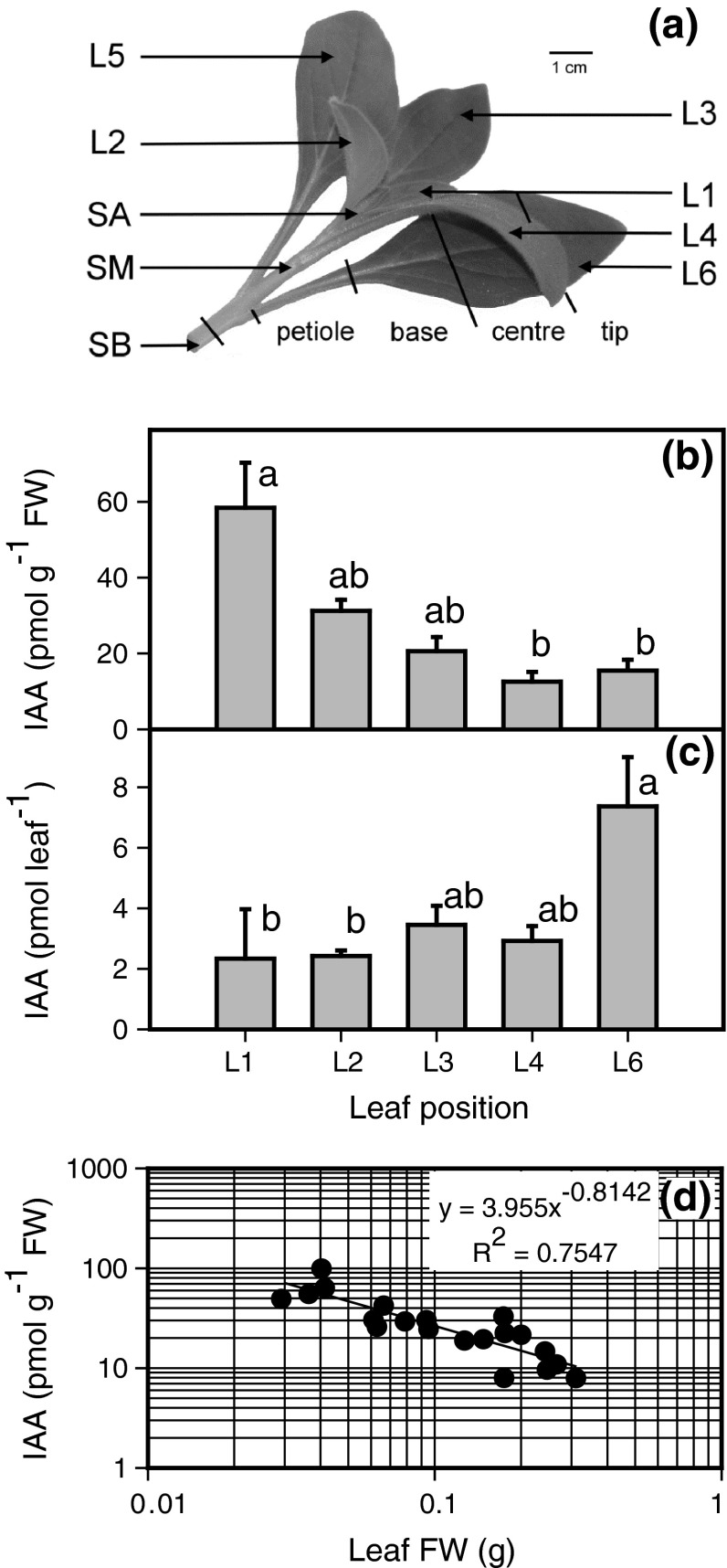
**a** Different sections of an excised leafy cutting of *P. hybrida* collected for IAA analysis. **b** IAA concentrations and **c** IAA contents in leaves (*L*) of different positions. **d** Relationship between leaf fresh mass and IAA concentration. *SA* shoot apex including smallest enclosing leaves; *SM* medium shoot position (1.5–2.5 cm from the basal end); *SB* stem base (0–0.5 cm). Mean and SE from five individual cuttings, columns which do not share a common letter are significantly different (*P* ≤ 0.05, Kruskal–Wallis test, *n* = 5). log log^−1^ plot of IAA concentrations versus leaf mass of 20 individual leaves (L1–L4) from five individual cuttings

Leafy stem cuttings of *Petunia hybrida* cv. Mitchell (original seeds provided from Marcel Buchers lab, ETH Zurich, Switzerland) were used for all experiments. Shoot tip cuttings (Fig. 1a) were excised from donor plants and were planted for rooting in plastic trays containing the chemically inert substrate perlite (‘Perligran A’, particle size 0–6 mm, Knauf Perlite GmbH, Dortmund, Germany). Growth conditions were as described in Ahkami et al. (2009). Foliar spraying of NPA (Duchefa, Haarlem, The Netherlands) was conducted immediately after excision of cuttings to inhibit basal auxin transport in the cuttings. NPA was dissolved in 1 ml 1 N NaOH according to the manufacturer’s protocol and diluted with distilled water to a final stock concentration of 100 μM. Appropriate volumes of stock solution were added to distilled water to obtain the different concentrations required. Five different concentrations of NPA, including 10, 25, 50, 80 and 100 μM, were tested. Consequently, the concentration of 50 μM NPA was used in the following experiments as it caused severe inhibition of rooting without causing wilting of the cuttings.

For analysis of the spatial distribution of IAA, samples of different plant tissues (complete leaves of five different positions, four transversal sections of lowest leaf and three stem sections, as illustrated in Fig. 1a) were immediately transferred into pre-weighed Eppendorf tubes, and the tubes were then re-weighed, immersed in liquid nitrogen and stored at −80 °C. For analysis of metabolites, enzyme activities and RNA extraction in the rooting zone, stem bases (0.5 cm) were abscised from non-treated and NPA-treated cuttings [no treatment within 0 h post excision (hpe)] at 11 time points (0, 2, 4, 6, 12, 24, 48, 72, 96, 144, 192 hpe) during rooting before any roots emerged and then sampled in the same way.

### Anatomical investigations and rating of rooting response

For histochemical examination of stem cuttings, 1-mm thick cross-sections were fixed overnight at 4 °C in 50 mM cacodylate buffer, pH 7.2, containing 2 % (v/v) glutaraldehyde and 2 % (v/v) formaldehyde, followed by one wash with buffer and two washes with distilled water. For secondary fixation, samples were transferred to a solution of 1 % (w/v) OsO_4_. After 1 h, samples were washed three times with distilled water. Dehydration at 21 °C was performed stepwise by increasing the concentration of ethanol (%, v/v) as follows: 30, 40, 50, 60, 75, 90 % and 2 × 100 % ethanol, for 1 h each. Samples were infiltrated with Lowicryl, LR White (Plano GmbH, Wetzlar, Germany) as follows: (%, v/v) 25 % overnight, 50 % and 75 % resin in 100 % ethanol for 4 h each, and then 100 % resin overnight. Samples were transferred into BEEM capsules, incubated there for 3 h in fresh resin, and polymerized at 60 °C for 48 h. Semi-thin sections (thickness 3 μm) were mounted on slides and stained for 2 min with 1 % (w/v) methylene blue and 1 % (w/v) Azur II in 1 % (w/v) aqueous borax at 60 °C before light microscopic examination, using a Zeiss Axiovert 135 (Jena, Germany) with an attached Zeiss Axiocam. After a rooting period of 14 days post excision, roots were counted, and then root length was determined as described in detail by Agulló-Antón et al. (2011).

### RNA isolation, northern blot analysis and Real-time PCR

Total RNA was extracted from *P. hybrida* cutting bases as described by Logemann et al. (1987). A Northern blot analysis was carried out as described by Ahkami et al. (2009). Four time points (0, 24, 72 and 144 hpe) were additionally analysed with Real-Time PCR. DNA was removed from RNA-extracts with RQ1 DNase (Promega, Madison, WI, USA), and first-strand cDNA was reverse transcribed using M-MLV RT RNase H reverse transcriptase (Promega) according to the manufacturer’s protocol. The relative cDNA abundance was detected by the i-cycler iQ (Bio-Rad, Hercules, CA, USA) using iQ SYBR Green SuperMix (Bio-Rad). The mRNA levels were determined by relative quantification using petunia actin mRNA sequence as a reference related to the 0 h control. The *Petunia* *GH3* gene (CV296522) was amplified from *Petunia* cDNA derived from the total RNA with the following specific primer pair (GH3 for: 5′-CACCGGCCCTTCAGTTCATC-3′; GH3 rev: 5′-CAGCAAGGCCACCAGGAGTC-3′), resulting in a fragment of 507 bp.

### Analysis of IAA, metabolites and enzyme activity

Extraction, clean-up and analysis of IAA were carried out according to a modified protocol as described by Müller et al. (2002). For extraction, 1 ml methanol containing 10 pmol (^2^H)_2_-IAA was added to a frozen sample together with five stainless steel balls (3 mm diameter) in a tube. The tube was immediately exposed to 60 °C for 20 min and the content subsequently disrupted for 20 min using a vibrating-ball micromill (Retsch MM301, Haan, Germany) at a vibration frequency of 30 s^−1^. After vortexing and incubation for 15 min at room temperature, the extract was centrifuged for 10 min at 14,000*g*, and the supernatant was collected. The residue was resuspended in 300 μl methanol, incubated and then centrifuged as described above. Pooled supernatants were reduced to dryness in a vacuum centrifuge (Savant SPD 111 V, Fisher Scientific, Schwerte, Germany) at 40 °C for 30 min at 320 mbar and thereafter at 200 mbar. The dried sample was dissolved in 50 μl methanol by vortexing and subsequent ultrasonic treatment for 5 min. After a short centrifugation, 200 μl diethyl ether was added, and the closed tube was vortexed and then exposed to ultrasonic treatment, followed by centrifugation, as described above. An aminopropyl solid phase extraction column (Chromabond NH_2_ shorty 10 mg, Macherey–Nagel GmbH, Düren, Germany) was equilibrated with 200 μl diethyl ether prior to application of the dissolved sample. The empty tube was flushed with 100 μl diethyl ether, which was also applied on the column. The column was washed twice with 200 μl diethyl ether, three times with 200 μl of a mixture of chloroform/2-propanol (2:1, v/v), three times with 200 μl chloroform and finally with 100 μl diethyl ether. The IAA fraction was eluted with three flushes of 200 μl diethyl ether containing 4 % acetic acid. Combined eluates were reduced to dryness in a stream of nitrogen at room temperature, redissolved in 20 μl methanol, methylated with 200 μl ethereal diazomethane, taken to dryness again and dissolved in 10 μl ethyl acetate.

Separation and mass fragment analysis were conducted using a Varian Saturn 2200 ion-trap mass spectrometer connected to a CP-3800 gas chromatograph (Agilent, Santa Clara, CA, USA) fitted with a CombiPal autoinjector (CTC Analytics AG, Zwingen, Switzerland).

The GC settings were as follows: splitless injection (1 μl) with 1-min pressure pulse at 24 psi; splitter opening 1:100 after 1 min; columns: Phe-Sil retention gap 10 m × 0.32 mm ID, ZB-50 50 % phenyl–50 % dimethylpolysiloxane 30 m × 0.25 mm ID, 0.25 μm film thickness, Phenomenex; carrier gas: He, 1 ml min^−1^, constant flow; temperature program: 1 min isothermally at 60 °C, followed by a linear ramp at a rate of 40 °C min^−1^ to 150 °C, isothermally for 6 min at 150 °C, followed by a linear ramp of 20 °C min^−1^ to 250 °C; transfer line temperature 230 °C.

The MS settings were as follows: CI-MRM mode; positive ion detection; reactant gas methanol; temperatures of manifold and ion trap 60 and 200 °C, respectively; axial modulation 4 V; scan time 0.4 s scan^−1^; multiplier offset 300 V; emission current 50 μA; maximum ionization time 2 ms; maximum reaction time 128 ms; waveform: resonant. Settings for endogenous IAA were chosen as follows (*m*/*z*): parent ion = 190 (M + H)^+^, diagnostic product ion = 130, excitation amplitude 0.5 V. A second channel analysing the isotopically labelled standard (^2^H)_2_-IAA used the parent ion (*m*/*z*) = 192 (M + H)^+^ and the diagnostic daughter ion (*m*/*z*) = 132. The amount of endogenous compound was calculated from the signal ratio of the unlabelled over the corresponding stable isotope-containing mass fragments. Recovery of the isotopically labelled standard was close to 50 %.

Carbohydrates were extracted in hot 80 % aqueous ethanol and glucose, fructose, and sucrose were analysed via enzymatic assay in microplates according to Hajirezaei et al. (2000). Amino acids were analysed in the same extracts after derivatization with 6-aminoquinolyl-*N*-hydroxysuccinimidyl carbamate by ultra-high performance liquid chromatography using fluorescence detection (excitation at 300 nm and emission at 400 nm) according to Zurbriggen et al. (2009). Extraction and determination of enzyme activities were conducted according to the method of Zrenner et al. (1995) with minor modifications, as described previously (Ahkami et al. 2009).

### Statistics

Because variances of data sets of IAA concentrations were not homogeneous among different groups according to the Hartley–Cochran–Bartlett test (*P* ≤ 0.05), differences between groups were tested using the Mann–Whitney *U* test for comparisons between two groups, and using the Kruskal–Wallis test for comparisons between several groups (*P* ≤ 0.05). Measurements of metabolites and enzyme activities were compared using the *t* test (*P* ≤ 0.05).

## Results

### Spatial distribution of IAA in *P. hybrida* cuttings

**Fig. 2:**
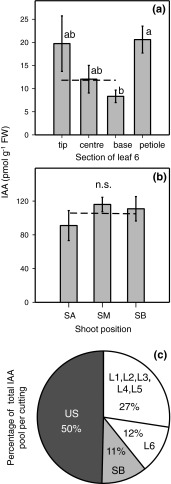
**a** IAA concentrations in transversal sections of L6 leaf. **b** IAA concentrations in different stem positions. **c** Total IAA pool sizes in different cutting parts of *P. hybrida*. *L* leaves of different positions, as shown in Fig. 1a. *US* complete upper stem above the stem base (*SB*); tip, leaf section 0–1.5 cm; centre, leaf section >1.5 cm and ≤3 cm; base, leaf section >3 cm. In **a** and **b**, mean and SE from five individual cuttings. Columns which do not share a *common letter* are significantly different (*P* ≤ 0.05, Kruskal–Wallis test, *n* = 5). *Dithered lines* indicate the mean IAA level in the complete lamina and shoot. IAA contents in **c** were calculated from IAA concentrations as illustrated in Fig. 1b and Fig. 2b (mean value of the three stem sections) and as estimated for L5 (14 pmol g^−1^ FW as mean value of L4 and L6) using the following fresh masses (mg): L1 (42), L2 (79), L3 (174), L4 (239), L5 (390), L6 (473), SB (65), US (290)

To elucidate the initial spatial distribution of IAA in the shoot tip cuttings and to assess the IAA pools possibly contributing to subsequent AR formation, first we analysed the IAA levels in different parts of cuttings at the time of excision from the donor plants. The different sections of an excised leafy petunia cutting are illustrated in Fig. 1a. Upon comparing leaves of different ages, IAA concentrations were highest in youngest leaves near the apex and decreased with lower position down to the fourth-eldest leaf (L4), the IAA level of which was similar to that of the lowest leaf (L6) close to the stem base (Fig. 1b). The L5 leaf was not analysed because its developmental stage was close to that of the L6 leaf. When focusing on the younger leaves, which showed the strongest variability in leaf size (L1–L4), we observed a strong inverse correlation between leaf weight and IAA levels. Thus, IAA concentrations followed a log log^−1^ plot versus leaf fresh mass, which explained 76 % of the variation in IAA concentration (Fig. 1d). Inverse to the gradient in IAA concentration (Fig. 1b), and strongly determined by leaf weight, the absolute pool size (content) of IAA among leaves was, however, highest in the lowest leaf (Fig. 1c). Transversal sections of L6 revealed significantly higher IAA concentrations in the petiole than in the attached leaf base (Fig. 2a). Variation of IAA level within the leaf lamina was not significant; the tip showed higher mean levels, but there was strong variation among individual samples. Stem tissues revealed highest IAA concentrations; levels were even higher than those found in younger leaves (Fig. 2b). However, there was no gradient of IAA level between the three stem sections. Calculating the absolute size of IAA pools in the different parts of the cuttings revealed that at the time of excision, about 40 % of IAA was located in the leaves, with 12 % in the eldest leaf and approximately 60 % in the stem tissue, with 11 % in the zone of subsequent root regeneration (Fig. 2c).

### Influence of NPA on AR formation in *P. hybrida* cuttings

**Fig. 3:**
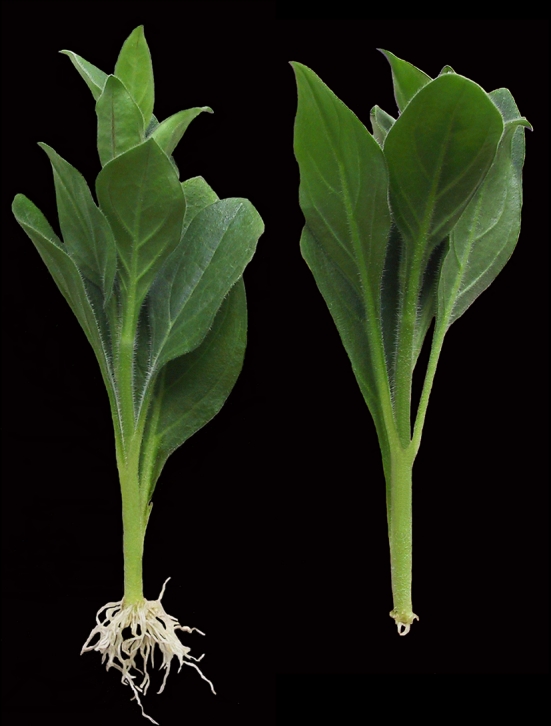
Picture of overall rooting of representative control (*left*) and NPA-treated (50 µM) cuttings of *Petunia hybrida* ‘Mitchell’ at 14 dpe. The background was digitally *blackened*

The auxin inhibitor NPA was employed to inhibit PAT and to evaluate the extent to which formation of ARs depends on basipetal transport from the upper shoot. Control cuttings showed intensive root formation. After 14 days, 96 % of cuttings were rooted and showed >50 roots per cutting (Table 1). Spraying of cuttings with NPA severely inhibited AR formation (Fig. 3). Thus, only 21 % of NPA-treated cuttings rooted until 14 days post excision, at very low intensity, as reflected by the low number of roots (Table 1). The overall rooting response as reflected by the total root length produced per planted cutting was reduced by the NPA treatment to 2 % of the value of the controls.

**Table 1:** Influence of NPA treatment (50 μM) on subsequent rooting of *P. hybrida* determined at day 14 post excision

	Percentage of rooted cuttings (%)	No. of roots per rooted cutting	Length per root (cm)	Total root length per planted cutting (cm)
Control	95.8 ± 4.2	53.2 ± 3.3	1.4 ± 0.06	47.7 ± 7.8
NPA	20.8 ± 4.2*	8.0 ± 2.9*	0.6 ± 0.04*	1.0 ± 0.4*

Mean values ± SE of four replications (each consisting of six cuttings)

* Significant effect of NPA application (*p* ≤ 0.05, *t* test or Mann–Whitney *U* test in case of total root length)

**Fig. 4:**
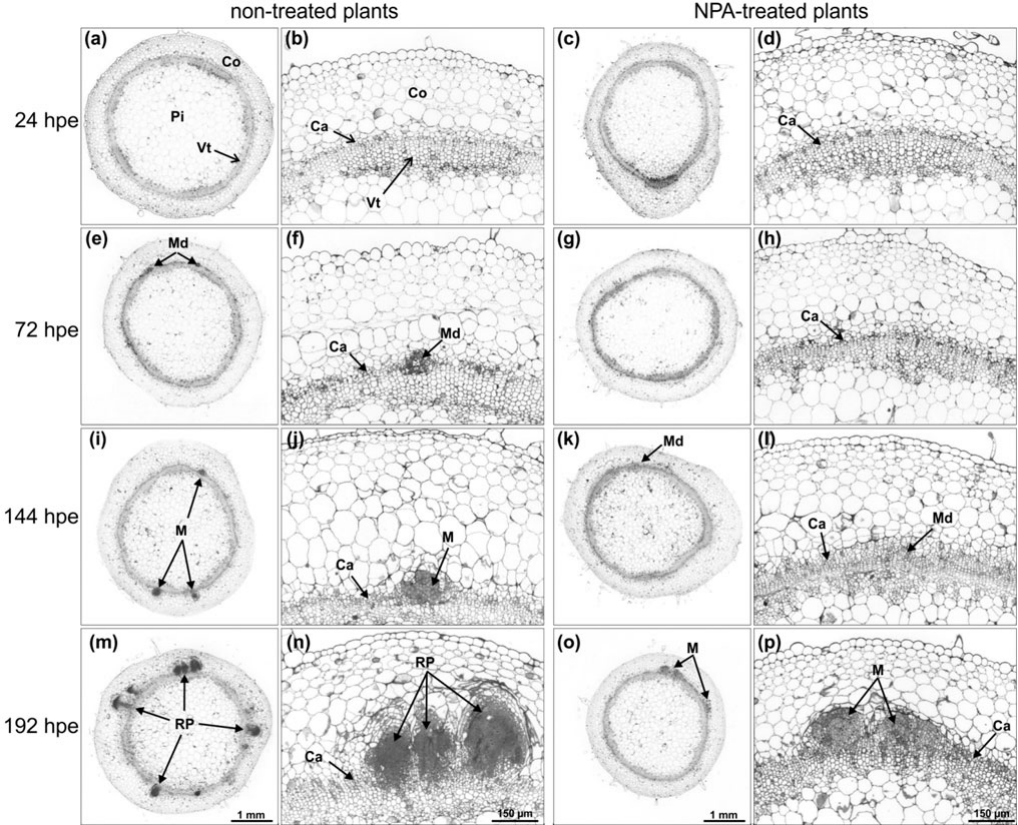
Light microscopy of stem base of *P. hybrida* cuttings during rooting under non-treated and NPA-treated conditions. In all micrographs, semi-thin cross-sections from 1 to 4 mm above the excision site are shown. **a**, **b** 24 hpe control cuttings. **c**, **d** 24 hpe NPA-treated cuttings. **e**, **f** 72 hpe control cuttings. **g**, **h** 72 hpe NPA-treated cuttings. **i**, **j** 144 hpe control cuttings. **k**, **l** 144 hpe NPA-treated cuttings. **m**, **n** 192 hpe control cuttings. **o**, **p** 192 hpe NPA-treated cuttings. *Ca* cambium, *Co* cortex, *M* meristem of AR, *Md* meristemoid of AR, *Pi* pith, *RP* primordia of AR, *Vt* vascular tissue, *hpe* hours post excision

The strong inhibition of AR formation in the cuttings in response to blocking of PAT was further highlighted by the anatomical studies. Control cuttings showed root meristemoids (new meristematic cells of the developing root meristems) at 72 hpe (Fig. 4e, f), globular root meristems at 144 hpe (Fig. 4i, j) and dome-shaped root primordia at 192 hpe (Fig. 4m, n). In NPA-treated cuttings, formation of new meristematic cells was not observed until 72 hpe (Fig. 4g, h) and was only hardly detectable at 144 hpe (Fig. 4k, l). In the same cuttings, few globular meristems appeared until 192 hpe when no dome-shaped primordia were observed (Fig. 4o, p).

### Temporal course of IAA during AR formation in response to NPA treatment

We then investigated the response of the IAA level in the rooting zone to the excision of cuttings and its dependency on PAT. Cuttings were planted, and the temporal course of IAA levels in the stem base of a non-treated control and of NPA-treated cuttings was monitored. Considering that oldest leaves contain the highest initial amount of IAA among the leaves (Fig. 1c) and according to the previous studies identifying fully developed leaves of carnation shoot tip cuttings as an important IAA source for subsequent AR formation (Garrido et al. 2002), we also monitored the IAA levels in the L6 leaves. Because those showed some variation of IAA levels within transversal sections (Fig. 2a), leaf discs (7 mm diameter), equally distributed between the tip, middle and base position of the leaf lamina, were included in each sample.

**Fig. 5:**
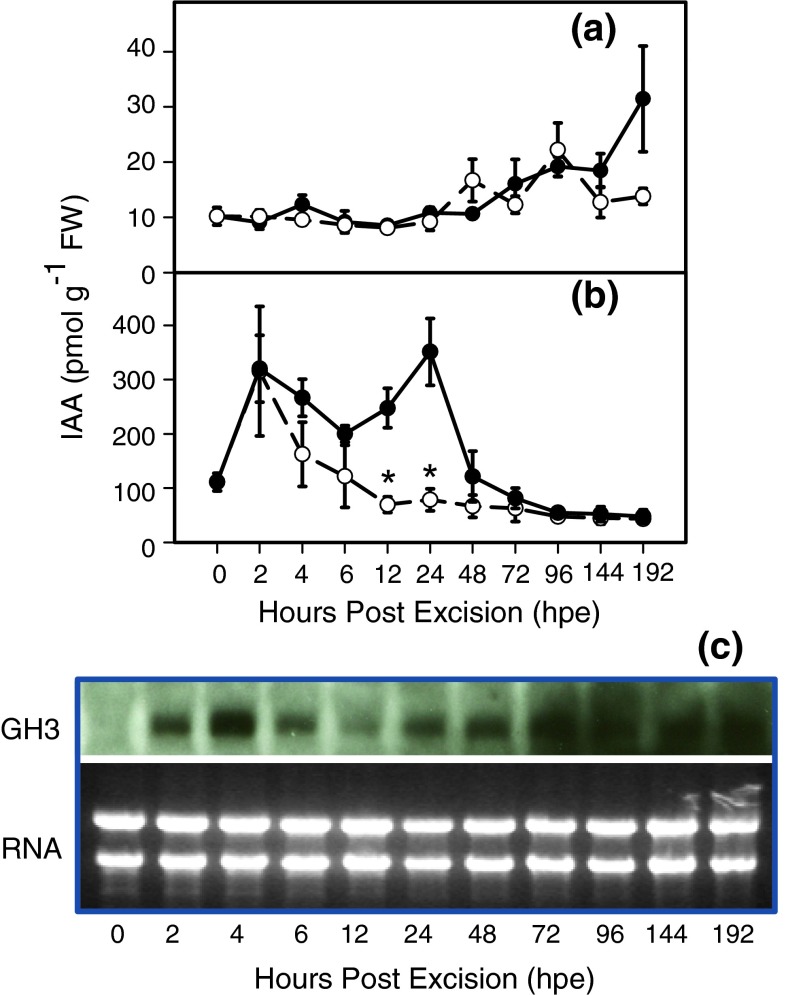
Temporal course of IAA concentrations in the lowest leaf (L6, **a**) and the basal stem (0.5 cm, **b**) of cuttings of *P. hybrida* during rooting under non-treated (*solid line*) and NPA-treated (*dashed line*) conditions (*n* = 5, each sample from two individual cuttings). *Asterisks* indicate a significant effect of the NPA treatment at the specified time after excision of cuttings (Mann–Whitney *U* test, *P* ≤ 0.05). **c** Transcript accumulation of auxin-responsive *GH3* gene in the basal stem of *P. hybrida* during rooting under standard (non-treated) conditions. Northern blot analysis was performed with 20 g RNA per sample, and separation on a 1.5 % (w/v) formaldehyde agarose gel. After transfer of RNA to a nitrocellulose membrane, it was hybridized to radioactively labelled cDNA fragments of the corresponding gene. The *below picture* shows the loading control of rRNA, the ratio of intensity of 28S RNA to 18S RNA (for total RNA) is 1:1

IAA levels in L6 leaves during the course of AR formation are illustrated in Fig. 5a. Control cuttings maintained initial IAA concentrations until 48 hpe, followed by a continuous rise until the end of the experiment. As a result, IAA concentrations at 96 and 192 hpe were significantly higher by 88 and 208 % compared with the initial level at the time of planting (*P* ≤ 0.05, Mann–Whitney *U* test, *n* = 5). After spraying with NPA, IAA levels followed a trend similar to that observed for the control until 144 hpe but with some fluctuation (Fig. 5a). In contrast to the control, NPA-treated cuttings did not accumulate IAA in L6 leaves until the end of the experiment.

With regard to the stem base (Fig. 5b), IAA concentrations in control cuttings significantly increased during the first 2 hpe. This was followed by a transient decrease until 6 hpe and a subsequent recovery to a maximum at 24 hpe. From 2 until 24 hpe, the stem base of control cuttings contained significantly higher IAA levels compared with those at the time of excision (*P* ≤ 0.05, Mann–Whitney *U* test, *n* = 5); at 24 hpe the initial IAA level was exceeded by +240 pmol g^−1^ fresh weight (FW) (+216 %). IAA concentrations decreased thereafter (Fig. 5b), and from 96 hpe onwards remained below the initial level measured at the time of planting (*P* ≤ 0.05, Mann–Whitney *U* test, *n* = 5). IAA in the stem base of NPA-treated cuttings exhibited the same increase until 2 hpe, as was observed for the control (Fig. 5b), but thereafter showed a sharp decrease to initial levels. In response to the NPA treatment, the IAA peak that was observed in the control cuttings was completely suppressed from 4 until 24 hpe (IAA levels at 4, 6, 12, 24 hpe no different from IAA level at 0 hpe, *P* ≤ 0.05, Mann–Whitney *U* test, *n* = 5). NPA-treated cuttings at 12 and 24 hpe showed significantly lower IAA levels in the stem base than in the control cuttings (Fig. 5b). After 72 hpe, NPA-treated cuttings showed the same low levels of IAA in the stem base as those observed for the control (Fig. 5b).

### Transcript accumulation of auxin-responsive *GH3* gene during AR formation

To determine whether the time course of IAA in the stem base of control cuttings is reflected by the expression of an auxin-responsive gene, the transcript levels of the *Petunia* *GH3* gene were monitored by Northern blot analysis. With a first increase to a maximum at 4 hpe, a decline thereafter, and a second increase beginning at 24 hpe (Fig. 5c), the *GH3* expression showed a corresponding pattern to the IAA levels in the rooting zone (Fig. 5b). The induction of *Petunia GH3* at 24 hpe and high expression at later stages (72 hpe, 144 hpe) were confirmed by quantitative Real-Time PCR (Online Resource 1).

### Influence of NPA on enzyme activities in the stem base

**Fig. 6:**
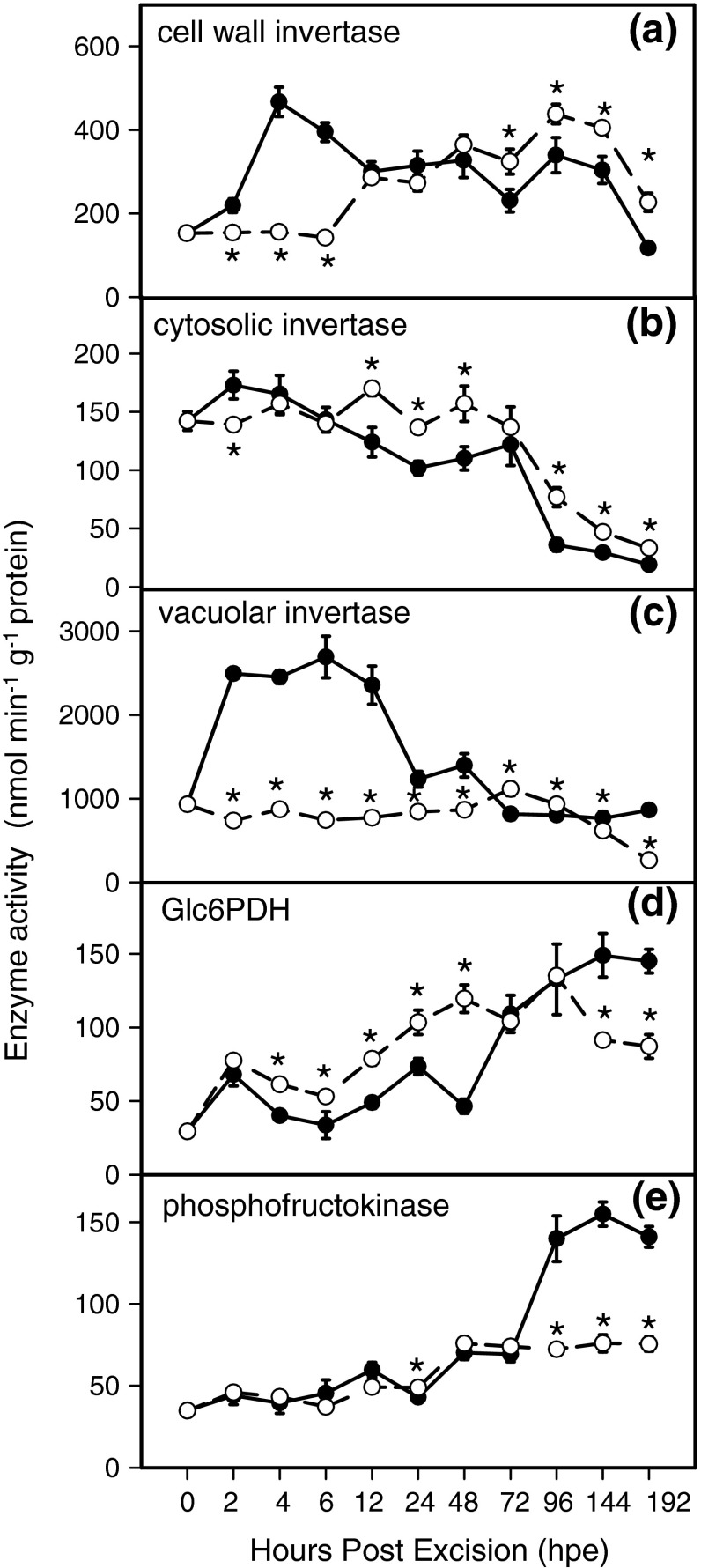
Alterations in the enzyme activities involved in sucrose degradation, glycolysis and pentose phosphate pathways in the basal stem of *P. hybrida* during rooting under non-treated (*solid line*) and NPA-treated (*dashed line*) conditions. **a** Cell wall invertase, **b** cytosolic invertase, **c** vacuolar invertase, **d** glucose-6-phosphate dehydrogenase, **e** phosphofructokinase. Each value is represented by the mean of five independent replicates ± SE. *Asterisks* indicate a significant effect of the NPA treatment at the specified time after excision of cuttings (*t* test, *P* ≤ 0.05)

To characterize further the effect of auxin transport on metabolic activity in primary metabolism during AR formation, the activities in the stem base of key enzymes involved in sucrose metabolism, the pentose phosphate pathway and glycolysis were analysed in response to NPA treatment. Results are illustrated in Fig. 6. In the non-treated cuttings, the activity of cell wall invertase increased soon after excision, peaking at 6 hpe, before decreasing to approximately its initial activity until 192 hpe. In contrast, cell wall invertase activity in NPA-treated cuttings remained at an initial low level until 6 hpe, and thereafter, increased up to twofold to levels similar to those of the control cuttings (Fig. 6a). The activity of cytosolic invertase showed a similar trend in both non-treated and NPA-treated cuttings, and decreased continuously during AR formation (Fig. 6b). The activity of vacuolar invertase increased up to two-and-half-fold in the stem base of non-treated cuttings and decreased until 192 hpe to reach approximately 30 % of the initial value. However, activity of vacuolar invertase in the stem base of NPA-treated cuttings remained unchanged until 96 hpe and decreased thereafter (Fig. 6c). Furthermore, in the stem base of non-treated cuttings, the activity of glucose-6-phosphate dehydrogenase (Glc6PDH) fluctuated in the first days of post excision and increased in the later course of AR formation (Fig. 6d). In NPA-treated cuttings, the activity of the same enzyme followed a similar trend; it was, however, less steep, with slightly higher activity than the control cuttings until 48 hpe, and vice versa after 96 hpe. The activity of phosphofructokinase (PFK) was similar and did not change significantly in the stem base of both non-treated and NPA-treated cuttings up to 72 hpe. Thereafter, PFK activity increased significantly in the stem base of non-treated cuttings up to 192 hpe, while it remained unchanged in the stem base of NPA-treated cuttings (Fig. 6e).

### Influence of NPA on sugar concentrations in the stem base

**Fig. 7:**
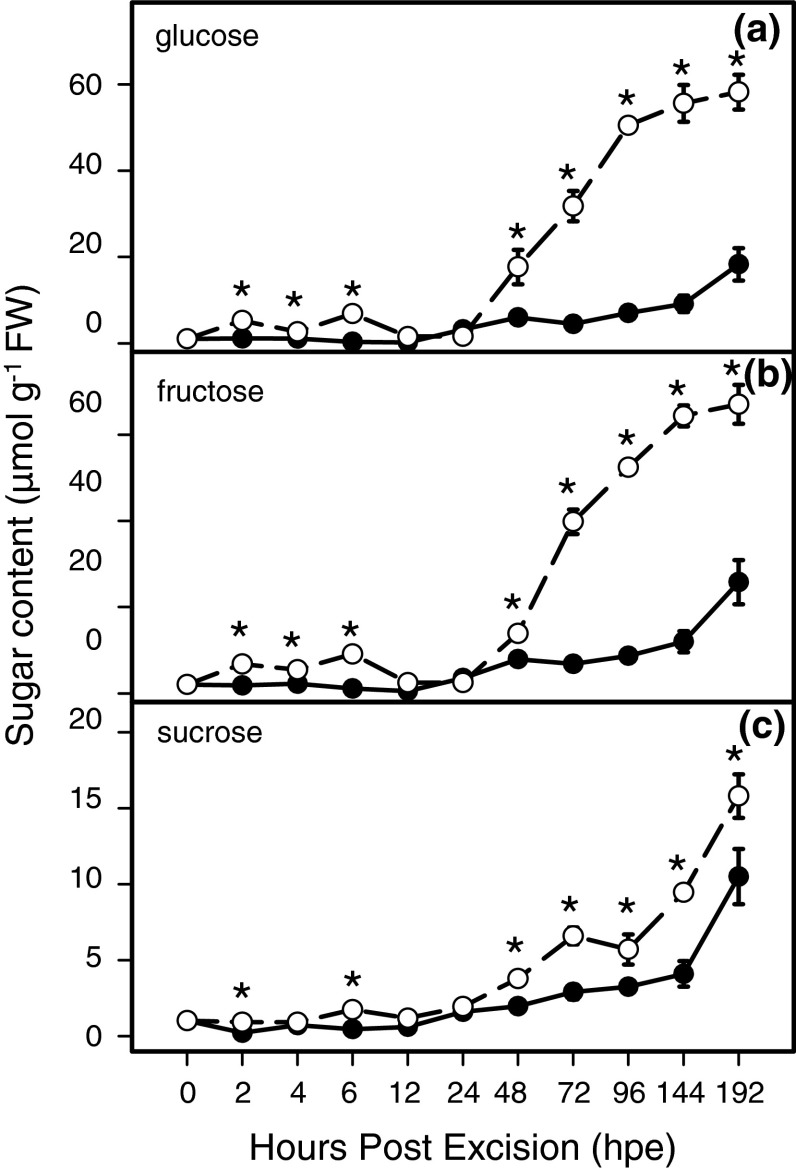
Concentrations of soluble sugars in the basal stem of *P. hybrida* during rooting under non-treated (*solid line*) and NPA-treated (*dashed line*) conditions. **a** Glucose, **b** fructose, **c** sucrose. Each value is represented by the mean of five independent replicates ± SE. *Asterisks* indicate a significant effect of the NPA treatment at the specified time after excision of cuttings (*t* test, *P* ≤ 0.05)

The concentrations of soluble sugars were measured during rooting in the stem base of non-treated and NPA-treated cuttings to evaluate whether auxin transport changes the distribution of the sugars. In the stem base of non-treated cuttings, a continuous increase of glucose, fructose and sucrose was observed after 24 hpe, reaching 10–15-fold higher levels at 192 hpe relative to the initial values (Fig. 7a–c). In the stem base of NPA-treated cuttings, the increase in concentrations of soluble sugars was even more pronounced. At later time points, levels were three times higher for glucose and fructose, and 1.5-fold higher for sucrose compared with the levels measured in the stem base of non-treated cuttings (Fig. 7a–c).

### Effect of NPA on soluble amino acids in the stem base

**Fig. 8:**
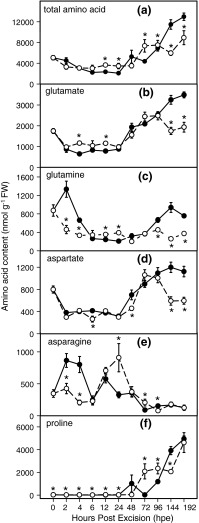
Concentrations of amino acids in the basal stem of *P. hybrida* during rooting under non-treated (*solid line*) and NPA-treated (*dashed line*) conditions. **a** Total amino acids, **b** glutamate, **c** glutamine, **d** aspartate, **e** asparagine, **f** proline. Each value is represented by the mean of five independent replicates ± SE. *Asterisks* indicate a significant effect of the NPA treatment at the specified time after excision of cuttings (*t* test, *P* ≤ 0.05)

To determine whether auxin transport changes the composition of soluble amino acids, which are necessary for protein synthesis, during AR formation, the concentrations of total and individual amino acids were determined in the stem base of both non-treated and NPA-treated cuttings. The concentration of total amino acids showed a similar trend in both non-treated and NPA-treated cuttings, and increased continuously after 24 hpe. However, the levels reached at 144 and 192 hpe were about 30–40 % lower in the stem base of NPA-treated cuttings compared with the controls (Fig. 8a). The concentrations of the most abundant amino acids in the stem base, including glutamate, glutamine, aspartate, asparagine and proline, were compared. Glutamate concentrations showed a trend similar to the total amino acids; at 144 and 192 hpe, concentrations reached a twofold higher level in non-treated cuttings compared with NPA-treated cuttings (Fig. 8b). Similar but even more pronounced differences were found for glutamine concentrations at 144 and 192 hpe (Fig. 8c). Furthermore, a threefold increase of glutamine concentration until 2 hpe was observed only in non-treated cuttings; it was absent in NPA-treated cuttings (Fig. 8c). The concentrations of aspartate, even though on a lower level, followed a similar trend and showed the same response to NPA treatment as glutamate (Fig. 8d). In the stem base of non-treated cuttings, the concentration of asparagine increased until 2 hpe and fluctuated thereafter during AR formation to decrease to a lower level than the initial value at 192 hpe. In contrast, the asparagine concentration in the stem base of NPA-treated cuttings decreased continuously up to 6 hpe but thereafter showed a transient increase to levels that were higher than those measured in non-treated cuttings (Fig. 8e). The concentration of proline increased constantly after 48 hpe, up to 200-fold at 192 hpe, compared with the initial values, in a similar manner for both non-treated and NPA-treated cuttings (Fig. 8f).

## Discussion

In the present study, the initial spatial distribution of IAA in *P. hybrida* shoot tip cuttings at the time of excision and the relationship between subsequent homeostasis of IAA in the rooting zone, PAT and AR formation were investigated. In addition, the interrelations between auxin and metabolic response in the rooting zone were elucidated.

### Spatial distribution of IAA in *P. hybrida* shoot tip cuttings before excision

We used GC–MS/MS to analyse the initial distribution of IAA in cuttings of *P.* *hybrida* at the time of excision. Concentrations found in the L3 leaves (Fig. 1b) and in the shoot apex (Fig. 2b) correspond to gas chromatography–mass spectrometry data published for similar tissues of the same cultivar (Tobeňa-Santamaria et al. 2002). The strong negative relationship established for young *Petunia* leaves between leaf fresh mass and IAA concentration (Fig. 1d) is in accordance with corresponding relationships found for *Arabidopsis*, where IAA levels were high in those leaves that showed high rates of cell division but strongly decreased when leaf expansion was initiated (Ljung et al. 2001). Ljung et al. (2001) also reported that for young leaves of *Nicotiana* that were in transition from division to elongation growth, high IAA levels were restricted to the base and middle of the blade as zones of intense cell division. We did not observe such a gradient, and the IAA levels were generally low in the lamina of *Petunia* L6 leaves (Fig. 2a), which had about 50 % of the maximum leaf length that we observed in old plants. This supports the assumption that cell division has almost completely ceased in L6 leaves. However, the higher IAA level in the petiole compared with the adjacent base (Fig. 2a) reflects the same accumulation pattern of IAA as observed in *Arabidopsis* and *Nicotiana* (Ljung et al. 2001; Müller et al. 2002), which indicates that active auxin transport occurred between the leaf and the stem of the *Petunia* cutting at the time of excision.

The similar IAA levels measured for the different stem positions of *P. hybrida* cuttings at the time of excision (Fig. 2b) are in accordance with data obtained for young shoots of other plant species (Nordström and Eliasson 1991; Kojima et al. 2002; Jager et al. 2007). It has been shown that auxin can be produced in, and exported from, diverse parts of the young shoot such as the apex, young leaves, and developing and also expanding leaves (Ljung et al. 2001; Garrido et al. 2002; Woodward and Bartel 2005; Jager et al. 2007). Thus, the same IAA levels for the lower and upper shoot positions may indicate a current auxin influx not only from the apex but also from the lower leaves of *Petunia* cuttings.

### The influence of PAT on auxin homeostasis and AR formation in *P. hybrida*

According to the current model, auxin travels through a combination of two processes: (1) rapid (5–10 cm h^−1^) non-directional transport that occurs in the vasculature and (2) slower PAT (5–10 mm h^−1^) (Friml and Palme 2002; Kerr and Bennett 2007). It has been further shown that conjunctions exist between these two routes. IAA from leaves can be loaded into the phloem, transported by this route (Morris and Kadir 1972; Borkovec et al. 1994) and then further transferred into the extravascular PAT pathway mainly at the shoot apex (Cambridge and Morris 1996). Specific influx [(auxin permease (AUX), like AUX (LAX)], specific efflux [pin-formed (PIN)], and multidrug resistance/P-glycoprotein [MDR/PGP] auxin carriers are involved, and their turnover, cycling and trafficking contribute to the asymmetric and dynamic nature of PAT (Morris et al. 2004; Kerr and Bennett 2007). The synthetic auxin transport inhibitor NPA is supposed to disrupt auxin efflux from the cell, even though the mode of action is still a matter of debate (Morris et al. 2004). It is thought that NPA acts via binding to a putative NPA-binding receptor protein, which is assumed to be located on the cytoplasmic face of the plasma membrane. It has been reported that inhibitors of PAT prevent the traffic of PIN1 and other rapidly cycled proteins to and from the plasma membrane in *Arabidopsis* root cells (Geldner et al. 2001). NPA has also been shown to interact with members of the MDR/PGB family in *Arabidopsis*.

The role of PAT in AR formation of cuttings has been studied intensively in carnations. Guerrero et al. (1999) measured the transport of labelled IAA through 1-cm long stem sections of freshly excised carnation cuttings. First and most of the labelled IAA passed the 1-cm section after a transport period of 1–2 h and 8 h, respectively, while application of NPA to the stem sections inhibited basipetal transport by 62–91 %, depending on the particular test conditions. Garrido et al. (2002) showed also that labelled IAA applied to mature leaves of cuttings was transported to the stem base and that excision of complete leaves, but not of the apex and of youngest leaves, inhibited rooting, while detached leaves could be partially substituted by auxin application to the stem. Application of NPA to the cuttings also resulted in inhibition of rooting in other plant species (Nordström and Eliasson 1991; Liu and Reid 1992; Koukourikou-Petridou and Bangerth 1997). In accordance to these studies, NPA severely inhibited and delayed the development of root primordia in *P. hybrida* (Figs. 3, 4) and decreased the overall intensity of rooting determined at 14 days post excision by 98 % (Table 1). A parallel analysis of IAA level highlighted the relationships between PAT, auxin accumulation in the rooting zone and different developmental phases in *Petunia* cuttings. The significant increase of IAA in the stem base of control cuttings between 2 and 24 hpe (Fig. 5b), which was followed by the formation of first meristematic cells at 72 hpe (Fig. 4e, f), contrasted with the elimination of the 24-h peak of IAA (Fig. 5b) and subsequent severe inhibition of adventitious rooting in NPA-treated cuttings (Figs. 3, 4; Table 1). This provides evidence for the essential contribution of PAT and the early accumulation of IAA in the rooting zone to spontaneous AR formation in *Petunia* cuttings in response to excision.

The kinetic of IAA levels in the rooting zone of control cuttings indicates two overlapping peaks, with maxima at 2 and 24 hpe, reflecting a strong regulation of auxin homeostasis soon after excision of *Petunia*. Similar fast changes of IAA levels have also been detected in the rooting zone of some other plant species (Blakesley et al. 1991). The observation that the 24-h peak of IAA was completely prevented by application of NPA provides evidence that this peak was the outcome of PAT. However, the first IAA maximum at 2 hpe was not prevented by NPA. We applied the NPA immediately after, but not before, excision of cuttings because we did not want to manipulate the initial auxin homeostasis in the cuttings before severance. Considering also the initial distribution of IAA pools within the cutting (Fig. 2c) and the speed of PAT, as discussed above, the missing effect of NPA until 2 hpe may reflect a lag phase between NPA application and its action on the cutting. NPA action may also have been underestimated by the IAA analyses of the complete stem (further discussed below). Nevertheless, the first IAA peak may also reflect transport in the phloem, local synthesis or mobilization from conjugates.

Wounding is an intrinsic process when cuttings are excised from donor plants, and we have already detected a strong increase of the wound-responsive hormone jasmonic acid in the stem base of *Petunia* cuttings during the first 30 min after excision (Ahkami et al. 2009). There are indications in the literature that wounding of plant tissues (Sztein et al. 2002) and jasmonates (Grsic et al. 1999; Sun et al. 2009) can stimulate IAA biosynthesis in plant tissues.

According to the inhibition of auxin efflux, it has been observed that auxins accumulate in cells treated with blockers and that application of NPA to stems stimulates auxin accumulation in the tissues above the NPA-treated region (Morris et al. 2004). Regarding the initial size of the IAA pool and the particular accumulation of IAA in the petiole of L6 leaves (Figs. 1c, 2a), we monitored the IAA level in L6 leaves as potential source organs for auxin export. As illustrated in Fig. 5a, IAA did not accumulate significantly in L6 leaves of NPA-treated cuttings compared with the non-treated ones. This provides no supportive indication of a predominant role of the L6 leaf as source organ for providing the auxin accumulation in the rooting zone. However, the observed oscillation of IAA levels in the NPA-treated cuttings may reflect a feedback inhibition of IAA biosynthesis in response to trapping of IAA, which has been found in expanding leaves of *Arabidopsis* (Ljung et al. 2001). Distinct blocking of auxin transport in separate parts of *Petunia* cuttings should be done in future studies, to elucidate these relationships. Interestingly, the IAA level showed a continuous increase after 72 hpe only in L6 leaves of non-treated cuttings; at 192 hpe, it reached a level significantly higher than the initial level (Fig. 5a). Considering that roots were formed in the non-treated cuttings during this period and regarding the possibility of de novo IAA biosynthesis in roots, which has been shown for *Arabidopsis* (Ljung et al. 2001), root-sourced auxin may have contributed to this increase.

### Auxin action and AR formation

We analysed the *Petunia* *GH3* gene expression in the stem base of non-treated control cuttings as an early marker of auxin activity (Hagen and Guilfoyle 1985) to obtain information on the involvement of auxin action at certain developmental stages of AR formation. The time course of *GH3* gene expression (Fig. 5c) followed the trend of the IAA level (Fig. 5b), while the strong increase of transcript level at 4 hpe indicated a lag phase of ca. 2 h behind the peaking of IAA. Considering the dose-dependent induction of the *GH3* promoter by active auxin, as shown in transgenic tobacco (Hagen et al. 1991; Li et al. 1999), these observations support the view that early auxin activity is causally involved in initiating the primary events of ARF in *Petunia.* Regarding also the fact that several *GH3* genes in *Arabidopsis* encode IAA-amido synthetases, which are important for maintaining auxin homeostasis by conjugating excess IAA to amino acids (Staswick et al. 2005; Wang et al. 2007), *GH3* expression may also contribute to auxin conjugation in *Petunia* cuttings to reduce the level of active auxin and thus to avoid inhibitory influences during later processes of AR formation (De Klerk et al. 1999). However, elucidation of these processes was not in the focus of the present study and requires further investigations involving quantification of IAA conjugates and *GH3* monitoring in response to NPA treatment.

Despite their broad use, the physiological mechanisms underlying the AR-induction by auxins are still far from being understood (Ludwig-Müller 2009). According to a recent model, plant phospholipase 2 is involved in auxin signal transduction during AR formation, and a large family of auxin-induced gene products (so-called ‘Aux/IAA’ proteins), auxin-responsive transcription factors (auxin response factors) and ARGONAUTE1 are involved in auxin-induced gene expression controlling the cellular events such as changes in cell cycle and cytoskeleton, tissue reorganization and cell wall modification (Ludwig-Müller 2009). Specific *GH3* genes may have particular functions in AR formation, which go beyond their role in auxin homeostasis, as supported by studies with *Arabidopsis* seedlings (Sorin et al. 2005). Recently, Gutierrez et al. (2012) showed that three auxin-inducible *GH3* genes are required for positive regulation of light-induced AR formation in *Arabidopsis.* They act by mediating the upstream interaction of three auxin response factors and downstream modulation of jasmonic acid homeostasis (Gutierrez et al. 2012).

Auxin might affect the root formation by acting directly in the cambium cells that initiate root primordia or indirectly through its engagement in the overall metabolism (Altman and Wareing 1975). Regarding the role of auxin in cell division and elongation, a complex interaction between auxin, cytokinin, cyclins and cyclin-dependent kinases (CDKs) governs the plant cell cycle (Hartig and Beck 2006; Komaki and Sugimoto 2012) Auxin treatment stimulated expression of cyclins including mitotic B1 cyclins in *Arabidopsis* (Ferreira et al. 1994; Richard et al. 2002), and expression of cyclin genes and of CDKs has already been related to auxin-induced AR formation (Lindroth et al. 2001; Neves et al. 2006). When *Petunia* cuttings experienced the same rooting conditions as applied to the controls in the present study, the transcript of a *Petunia CycB1* encoding a mitotic B1 cyclin accumulated in the stem base at 48 hpe (Ahkami et al. 2009). Based on the observation of IAA accumulation until 24 hpe (Fig. 5b), this may have contributed to the induction of Petunia *CycB1* and thereby stimulated the initiation of cell division. Furthermore, other genes such as SCARECROW-like genes may be involved; expression has already been shown to be stimulated in rooting-competent cuttings of *Pinus radiata* and *Castanea sativa* by auxin treatment before activation of cell division (Sánchez et al. 2007).

### Involvement of PAT and auxin homeostasis in the response of primary metabolism during AR formation

Plant meristems are utilization sinks, in which cell division activity governs sink strength (Hartig and Beck 2006). Monitoring of enzyme activities in the stem base of *P. hybrida* cuttings during AR formation revealed that some enzymes, such as cell wall and vacuolar invertases, as well as PFK, were modulated at specific root developmental stages and that these modulations were subjected to NPA treatment (Fig. 6). While the activity of invertases showed a maximum during the first hours of post excision in non-treated cuttings, the early increases of cell wall invertase (Fig. 6a) and of vacuolar invertase (Fig. 6c) were prevented by NPA application, indicating that PAT contributed to the increase in activities of these sucrose degrading enzymes. These results reflect a specific relationship between basipetal auxin transport and extracellular and vacuolar invertases, and suggest that auxin may regulate the activity of enzymes in the sink establishment phase of AR formation in *Petunia* cuttings. Importantly, one of the proposed functions of cell wall invertase is to act as a linker between hormonal responses and primary metabolism (Roitsch and Gonzalez 2004). Even though there is indication in literature that vacuolar and cell wall invertases can be stimulated by auxin (Morris and Arthur 1984, Lee et al. 1997), the underlying mechanisms are far from being understood. The dominant mechanism that determines steady-state level of invertases is a highly responsive transcriptional regulation; however, invertases are further controlled at post-translational level and via protein trafficking and transcript turnover (Roitsch and Gonzalez 2004; Albacate et al. 2011). Yun et al. (2002) could counteract a rapid repression of vacuolar invertase in excised etiolated hypocotyl segments of mung bean by incubation with IAA, while the auxin treatment increased enzyme activity and the level and half-life of transcripts of the vacuolar invertase gene *VR*-*AI1*. Responses to inhibitors of polymerase II and of protein biosynthesis further supported the view that IAA enhanced transcript stability. Proels et al. (2003), who described the full genomic sequence and expression pattern of the *LIN5* gene coding for an extracellular invertase in tomato, provided evidence that auxin stimulated expression of this gene while auxin responsiveness was mediated via a 1.6-kb *Lin5* promoter fragment.

The temporal courses of IAA and invertase activities in the stem base reveal that NPA treatment-reduced activities of cell wall (4 hpe, Fig. 6a) and of vacuolar invertases (2 hpe, Fig. 6c) before a significant reduction in IAA level were detected (12 hpe, Fig. 5b). PAT in stems occurs in the xylem parenchyma cells, the vascular cambium and its partially differentiated derivative (Gälweiler et al. 1998; Morris et al. 2004). Thus, IAA might have responded earlier to the NPA treatment in particular tissues and cell compartments, and thereby influenced invertase activity before a significant change of IAA level was detected in the complete stem base. Future studies of spatial and cellular distribution of auxin activity by use of auxin-responsive promoters in response to NPA treatment may elucidate these relationships.

Comparing enzyme activities with measured sugar levels, it has to be considered that activities of invertases were determined for certain cell compartments (apoplast, cytosol, vacuole), whereas sugar levels reflect the mean situation of the whole stem base of the cuttings. Furthermore, sugar levels may be additionally influenced by fluxes or metabolic events which are not limited by those enzymes analysed in this study. Considering the period between 4 and 12 hpe, NPA treatment decreased the activities of cell wall and vacuolar invertases but slightly enhanced the levels of glucose and fructose. Apart from the influence of cell compartments, this may indicate that NPA also inhibited utilisation of hexoses. As already discussed above, the expression of mitotic cyclin genes is typical for cells shortly before they divide (Ferreira et al. 1994), and an enhanced expression of a *Petunia CycB1* gene was detected in *P. hybrida* at 48 hpe when rooting conditions were the same as applied to the control cuttings in the present study (Ahkami et al. 2009). Considering, that replication of DNA, RNA and protein synthesis occurs before the mitosis phase of the cell cycle (Hartig and Beck 2006), there is a need of carbon skeletons before cell division starts. In conclusion, the slightly higher sugar levels in NPA-treated cuttings compared to the controls between 4 and 12 hpe may reflect a higher ratio between hexose consumption due to auxin-stimulated cell division on the one side and sucrose delivery and hexose release on the other side. However, the data of Glc6PDH and of PFK do not provide indication that these enzymes were primarily bottlenecks of glucose utilization during this early phase. Although subsequent root formation was delayed using the blocker of PAT (Fig. 4), soluble sugars, particularly, hexoses accumulated more strongly in the stem base of NPA-treated cuttings beginning 48 h after excision compared with the controls (Fig. 7). From a first point of view, these results seem to contradict findings that auxin application to the stem base of cuttings of other plant species enhances the sugar level in the rooting zone (Altman and Wareing 1975; Husen and Pal 2007; Agulló-Antón et al. 2011). However, it has to be taken into account that in the present study, the role of “natural auxin level” in response to excision of cuttings was investigated. In the studies mentioned above, applied auxin probably enhanced auxin concentration in the rooting zone to much higher levels so that the balance between the responses at sink establishment level (modifying carbohydrate influx) versus root development level (modifying carbohydrate utilization) might have been different in our study. Based on our observations, we conclude that the elevated sugar accumulation in NPA-treated *Petunia* cuttings during the later stages of AR formation is not directly affected by the lower auxin level until 24 hpe. This phenomenon is rather the outcome of the lower utilization of carbohydrates due to the strongly inhibited root formation. This conclusion is strongly supported by our finding that during the period after 96 hpe, the NPA-treated cuttings demonstrated significantly lower activities of PFK and of Glc6PDH than the control cuttings.

Amino acids are needed during AR formation because they provide essential components for protein synthesis required for cell division, elongation and function. In accordance with findings of earlier studies (Ahkami et al. 2009), total amino acids, glutamate, glutamine and aspartate accumulated in the rooting zone of control cuttings in this study as well. The highest levels were observed during the period following 96 hpe (Fig. 8), when root meristems and primordia were formed (Fig. 3). Accumulation of those amino acids was significantly lower in NPA-treated cuttings (Fig. 8b–d). Interestingly, inhibition of AR formation was associated with increased sugar levels but with decreased amino acid levels, even though both can be considered as important resources for root development (Haissig 1986). This discrepancy suggests that the intensity of influx and biosynthesis of amino acids in the rooting zone is directly related to differentiation and growth of ARs (Fig. 3), whereas the sugar level in the rooting zone, which is also highly dependent on the strength of the carbon source in the cutting (Rapaka et al. 2005), reflects the balance between influx and utilization of carbon. In this context, *Petunia* cuttings reveal high carbon assimilation, comparable to that of intact donor plants even at irradiance lower than that used in the present study (Klopotek et al. 2012).

Interestingly, NPA treatment led to inhibition of the early increase of glutamine and asparagine in the rooting zone in the sink establishment phase (Fig. 8c, e). These two compounds belong to the group of amino acids transported in the phloem (Urquhart and Joy 1982; Lohaus and Moellers 2000), and asparagine is suggested to be the main nitrogen transport compound in the initiating roots of cuttings (Suzuki and Kohno 1983). Considering on the one hand, the possible roles of PAT and early auxin accumulation (Fig. 4b) in driving sucrose utilization via stimulation of cell wall and vacuolar invertase (Fig. 6a, c), and on the other hand the mechanism of co-transport of sucrose and amino acids in the phloem (Lalonde et al. 2004), the lower levels of both amino acids in the stem base of NPA-treated cuttings may be the result of decreased apoplastic unloading of sucrose.

A strong accumulation of proline was found at later stages of AR formation, while similar levels were monitored in both non-treated and NPA-treated cuttings (Fig. 8f). These results do not support a role of PAT or of auxin homeostasis in proline accumulation during AR formation in *Petunia* cuttings. This is in contrast with the observations that auxin application increased the levels of proline in mung bean hypocotyls (Kuraishi et al. 1967) and transcripts of proline-rich proteins (PRPs) involved in lateral root formation in *Arabidopsis* and carrot (Ebener et al. 1993; Neuteboom et al. 1999; Bernhardt and Tierney 2000). However, it has been suggested that auxin acts as a regulator of PRP genes in a particular time- and concentration-dependent manner (Thomas et al. 2003).

## Conclusive model

**Fig. 9:**
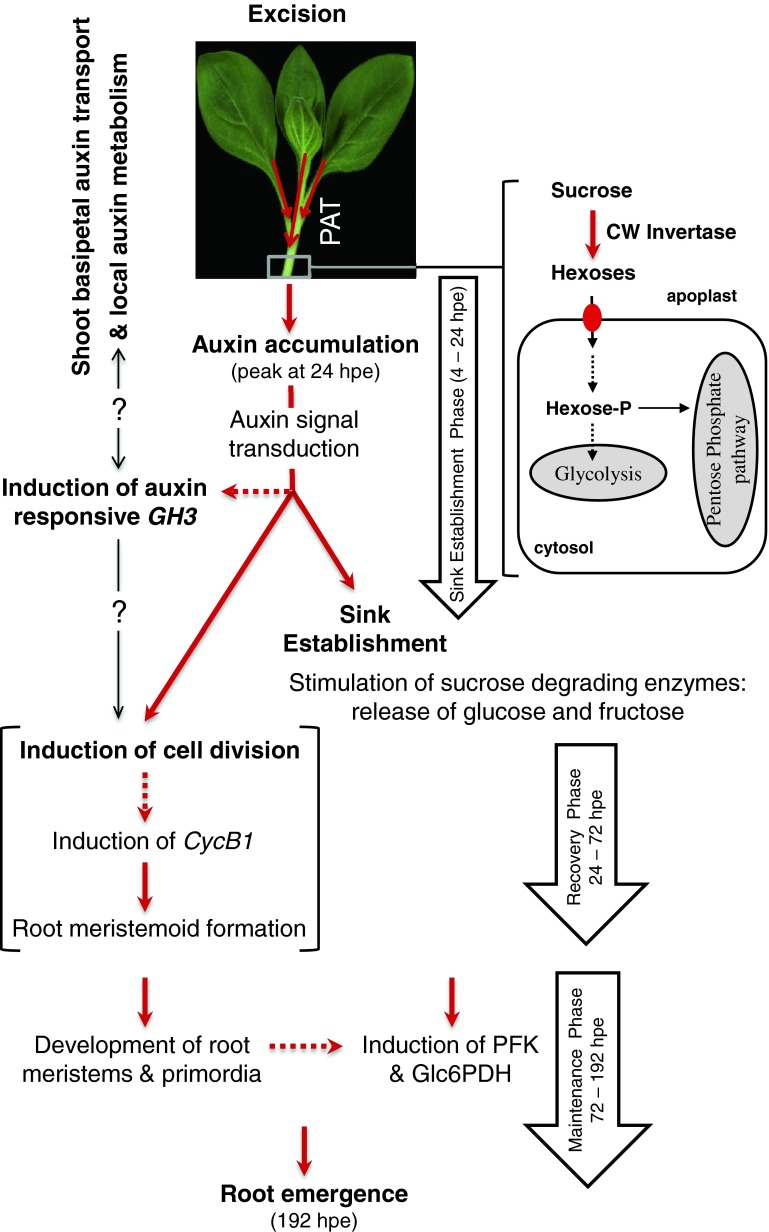
A postulated model of relationship between PAT, auxin accumulation, primary metabolism and AR formation in *Petunia* cuttings in response to excision from the donor plant. *Red solid arrows* indicate processes that are dependent on PAT and are correlated with the resulting auxin peak at 24 hpe in the rooting zone. *Red dashed arrows* indicate additional processes which are hypothetically depending on PAT as supported by data of the present study and of Ahkami et al. (2009). *Thin black arrows* with question mark show possible interrelations between *GH3* induction, auxin metabolism and cellular events of AR formation. Further details regarding different stages and phases of AR formation in petunia cuttings are described in Ahkami et al. (2009). *PAT* polar auxin transport, *CW* invertase cell wall invertase, *PFK* phosphofructokinase, *Glc6PDH* glucose-6-phosphate dehydrogenase, *hpe* hours post excision

Overall, our study clearly shows that spontaneous AR formation in leafy stem cuttings of *P. hybrida* is dependent on PAT and on resulting auxin homeostasis in the rooting zone. A respective model of the relationship between PAT, auxin, primary metabolism and AR formation is postulated and illustrated in Fig. 9. After excision of cuttings, PAT enables the accumulation of free auxin in the rooting zone, where it (1) contributes to the establishment of the new sink via stimulation of cell wall and vacuolar invertases and (2) induces the first cell divisions of the new root meristems. After PAT-dependent induction of root meristems and sink establishment, the decrease of auxin levels allows for subsequent root development, which in turn stimulates the entry of glucose into glycolysis and the pentose phosphate pathway as reflected by the NPA-inhibited activation of PFK and G6PDH. Stimulated glycolysis and pentose pathway generate the energy and carbon skeletons to be used for synthesis of amino acids, proteins and nucleic acids. The present data further support the assumption that accumulation of free auxin contributes to induction of *GH3* gene while previous results (Ahkami et al. 2009) suggest involvement of Petunia *CycB1* gene in induction of cell division. GH3 may stimulate auxin conjugation thereby reducing the level of active auxin after 24 hpe and may be further involved in initiation of cellular events. Activity of sucrose degrading enzymes provides the hexoses needed for supply of energy and of carbon to cell proliferation, differentiation and growth. Considering the effects of sugars, particularly of hexoses on gene expression (Rolland et al. 2006), released hexoses may be further involved in the control of cell cycle (Hartig and Beck 2006) and modification of auxin homeostasis (Sairanen et al. 2012) and signal transduction (Mishra et al. 2009), while the steady-state levels of individual sugars reflect the balance between the influx and degradation of sucrose and the utilization of hexoses via glycolysis and pentose phosphate pathway.

## Electronic supplementary material

Below is the link to the electronic supplementary material.

Supplementary material 1 (PDF 323 kb)

## Abbreviations

AR: Adventitious root
AUX: Auxin permease
CDK: Cyclin-dependent kinase
FW: Fresh weight
GC–MS/MS: Gas chromatography–tandem mass spectrometry
GH3: Gretchen Hagen 3
Glc6PDH: Glucose-6-phosphate dehydrogenase
Hpe: Hours post excision
IAA: Indole-3-acetic acid
IAAasp: Indole-3-acetylaspartic acid
LAX: Like AUX
MDR: Multidrug resistance
PGP: P-glycoprotein
NPA: Naphthylphthalamic acid
PAT: Polar auxin transport
PFK: Phosphofructokinase
PIN: Pin-formed
PRP: Proline-rich protein
TIBA: Triiodobenzoic acid
