# Supplementary material for: Distribution of indole-3-acetic acid in Petunia hybrida shoot tip cuttings and relationship between auxin transport, carbohydrate metabolism and adventitious root formation
Source: Planta. 2013 Jun 14;238(3):499–517. doi: 10.1007/s00425-013-1907-z (PMC3751230; doi:10.1007/s00425-013-1907-z)
Supplement: Supplementary file 1 — Supplementary material 1 (PDF 323 kb) [file 425_2013_1907_MOESM1_ESM.pdf]

Planta

ORIGINAL ARTICLE

Distribution of indole-3-acetic acid in *Petunia hybrida* shoot tip cuttings and relationship between auxin transport, carbohydrate metabolism and adventitious root formation

Amir H. Ahkami, Michael Melzer, Mohammad R. Ghaffari, Stephan Pollmann, Majid Ghorbani Javid, Fahimeh Shahinnia, Mohammad R. Hajirezaei and Uwe Druege

Corresponding authors:
Uwe Druege, Leibniz Institute of Vegetable and Ornamental Crops, Erfurt, Germany, e-mail: druege@erfurt.igzev.de;
Mohammad-Reza Hajirezaei, Leibniz Institute of Plant Genetics and Crop Plant Research (IPK), Gatersleben, Germany, email: mohammad@ipk-gatersleben.de

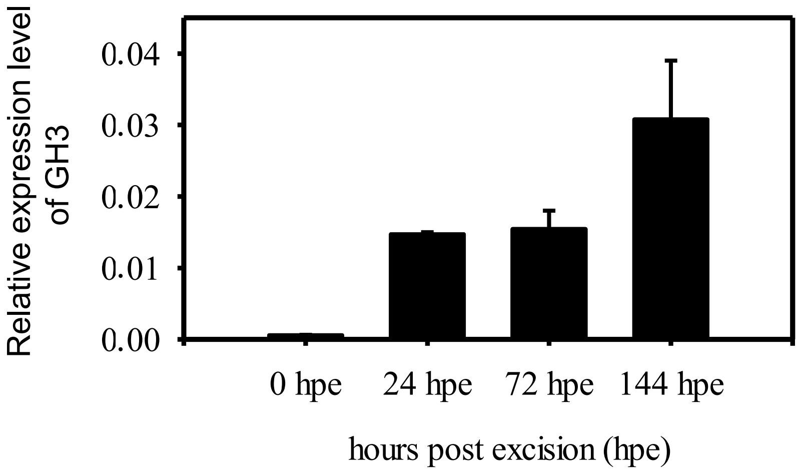

Online Resource 1. Quantitative Real-Time PCR of *Petunia GH3* during adventitious root formation in cuttings of *Petunia hybrida* 'Mitchell'. Three independent biological replicates for each time point.
